# Impact of Co-Existing Placental Pathologies in Pregnancies Complicated by Placental Abruption and Acute Neonatal Outcomes

**DOI:** 10.3390/jcm10235693

**Published:** 2021-12-03

**Authors:** Dorsa Mavedatnia, Jason Tran, Irina Oltean, Vid Bijelić, Felipe Moretti, Sarah Lawrence, Dina El Demellawy

**Affiliations:** 1 Department of Medicine, University of Ottawa, Ottawa, ON K1H 8M5, Canada; dmave060@uottawa.ca (D.M.); trn.jason@gmail.com (J.T.); fmoretti@toh.ca (F.M.); 2 Children’s Hospital of Eastern Ontario Research Institute, Ottawa, ON K1H 8L1, Canada; IOltean@cheo.on.ca (I.O.); VBijelic@cheo.on.ca (V.B.); 3 Department of Pathology, Children’s Hospital of Eastern Ontario, Ottawa, ON K1H 8L1, Canada

**Keywords:** infant, newborn, female, pregnancy, abruptio placentae, Apgar score, pregnant women, gestational age, placenta

## Abstract

Placental abruption (PA) is a concern for maternal and neonatal morbidity. Adverse neonatal outcomes in the setting of PA include higher risk of prematurity. Placental pathologies include maternal vascular malperfusion (MVM), fetal vascular malperfusion (FVM), acute chorioamnionitis, and villitis of unknown etiology (VUE). We aimed to investigate how placental pathology contributes to acute neonatal outcome in PA. A retrospective cohort study of all placentas with PA were identified. Exposures were MVM, FVM, acute chorioamnionitis and VUE. The primary outcome was NICU admission and the secondary outcomes included adverse base deficit and Apgar scores, need for resuscitation, and small-for-gestational age. A total of 287 placentas were identified. There were 160 (59.9%) of placentas with PA alone vs 107 (40.1%) with PA and additional placental pathologies. Odds of NICU admission were more than two times higher in pregnancies with placental pathologies (OR = 2.37, 95% CI 1.28–4.52). These estimates were in large part mediated by prematurity and birthweight, indirect effect acting through prematurity was OR 1.79 (95% CI 1.12–2.75) and through birthweight OR 2.12 (95% CI 1.40–3.18). Odds of Apgar score ≤ 5 was more than four times higher among pregnancies with placental pathologies (OR = 4.56, 95% CI 1.28–21.26). Coexisting placental pathology may impact Apgar scores in pregnancies complicated by PA. This knowledge could be used by neonatal teams to mobilize resources in anticipation of the need for neonatal resuscitation.

## 1. Introduction

Placental abruption remains a critical concern for maternal, fetal, and neonatal morbidity and mortality [1,2]. In fact, it is a rare but serious complication affecting 3 to 10 per 1000 pregnancies worldwide [3], accounting for around 10–20% of all neonatal deaths in developed countries [4]. Maternal complications associated with placental abruption include hemorrhagic shock, disseminated intravascular coagulation (DIC), kidney failure, organ ischemia and necrosis, and death, related to coronary heart disease and stroke [5,6]. Adverse neonatal outcomes in cases of severe placental abruption, include higher risk of fetal-growth restriction, stillbirth, prematurity, and birth asphyxia [7,8,9,10,11]. However, not all cases of abruption result in poor neonatal outcomes [12]. In the context of placental abruption, obstetricians may face difficulties in deciding when to intervene and deliver. Typically, in severe placental abruption cases, emergent cesarean section with shorter onset-to-delivery time is recommended, to prevent intrauterine fetal death, neonatal morbidity and mortality [13]. In addition to onset-to-delivery time, obstetricians must weigh the severity and chronicity of placental abruption and the mother’s clinical status with the gestational age of the fetus [13,14,15] in deciding the best timing for delivery to optimize obstetric and neonatal outcomes. The potential impact of maternal underlying placental pathologies in cases of PA may help contribute to the decision making. 

Underlying placental pathologies include maternal vascular malperfusion (MVM), fetal vascular malperfusion (FVM), acute chorioamnionitis, and villitis of unknown etiology (VUE) [16]. These pathologies are independently associated with the presence of neonatal complications [17,18,19,20]. In particular, MVM is prevalent in cases of hypertensive disorders of pregnancy [21], fetal-growth restriction [22], preeclampsia [23], and is highly associated with preterm labour and premature rupture of membranes [24,25,26,27]. Recent literature also suggests that pregnancy complications can recur in subsequent pregnancies, regardless of the presence of MVM lesions [28]. Moreover, severe onset of FVM can increase the chance of fetal growth restriction, fetal central nervous system (CNS) damage and neurodevelopmental delays, and stillbirth [29,30]. Chorioamnionitis can result in postpartum uterine infections [31,32], fetal death and neonatal sepsis [33,34], intraventricular hemorrhage (IVH), asphyxia, and cerebral white matter damage [35,36,37]. VUE can impede fetal growth and contribute to recurrent reproductive loss [38]. 

The aim of this present study is to investigate if placental pathologies can adversely affect acute neonatal outcome in pregnancies complicated with PA. Our hypothesis is that additional placental pathologies will deplete placenta reserve, impair placental function prior to any abruption event, and thus contribute adversely to neonatal outcome. This is knowledge needed to inform best clinical practice for obstetricians in Canada. 

## 2. Material and Methods

### 2.1. Data Collection

A retrospective cohort study was conducted at The Ottawa Hospital (TOH) and the Children’s Hospital of Eastern Ontario (CHEO). All placentas with the pathologic or clinical diagnosis of placental abruption and/or retroplacental hematoma from 1 October 2013 to 30 April 2020 were identified from the pathology archives using the Laboratory Information Service Program (EPIC-Hyperspace) [39]. Data on maternal demographics, neonatal outcomes, and the gross and histopathological findings of the placenta were collected using EPIC-Hyperspace and recorded on RedCap [40]. The clinical diagnosis of abruption was defined as either Obstetric ultrasound with direct visualization of subchorionic or retroplacental hematoma or clinical presentation of vaginal bleeding, abdominal pain, uterine contractions and/or uterine tenderness, as defined on the TOH electronic medical records (EMRs). The pathological diagnosis was then confirmed post-delivery, through the CHEO EPIC report, using the reports on placental examination for the presence of retroplacental clot(s). A total of 287 placentas were identified. Placentas were excluded if maternal-neonatal linked charts could not be located (*n* = 26) or there were missing data on study outcomes (percent missing ranged from 18 % to 28%). Institutional approval was obtained prior to study initiation (CHEOREB# 20/22X). 

We divided the dataset into clinically diagnosed placental abruption only (presence of adherent retroplacental clot and typical findings defined above) versus placental abruption with any placental pathologies of MVM, FVM, acute chorioamnionitis, and VUE. The criteria for diagnosing placental lesions followed the 2016 Amsterdam consensus statement [16]. Of note, we considered the presence of any independent lesion external from the section taken in the area of placental abruption. For there to be a definitive MVM diagnosis, accelerated villous maturation had to be present in all cases. More importantly, infarction in the section of abruption alone is not taken as a sign of MVM. Further details regarding the definitions for each lesion are found in the Supplementary Materials (S1). Our primary outcome was NICU admission, while the secondary outcomes included BD 10–15.9 or BD ≥ 16, cord pH ≤ 7 or 7.1–7.15, Apgar score ≤ 5 at 10min, need for resuscitation, and small-for-gestational age (SGA). 

### 2.2. Statistical Analysis 

Demographic and anthropometric characteristics were summarised descriptively with median and interquartile range (IQR) for continuous variables, and frequencies and percentages for categorical variables and compared between the group with co-occurring placental lesions) versus the group of placental abruption only, using the Wilcoxon rank sum for continuous variables and Fischer’s exact test for discrete variables. The effect of placental pathology on NICU admission was investigated using unadjusted and adjusted logistic regression. Directed acyclic graph (DAG) [41] was constructed to guide the selection of confounding and mediating variables and to inform the final multivariable analysis of the primary outcome, NICU admission. The extent to which placental lesions contribute to NICU admission was assessed using the causal mediation analysis with prematurity and birthweight as mediators. The effect of each mediator was analyzed separately. We considered the exposure-mediator interaction between placental pathology and prematurity (GA < 37 weeks) and interaction between placental pathology and birthweight. Interactions were not significant and were removed from the final mediation analysis. Both mediation analyses were adjusted for maternal smoking, maternal hypertension/preeclampsia, and maternal diabetes. The effect of placental pathology on secondary outcomes were investigated using unadjusted logistic regression. Two-sided *p* values less than 0.05 and odds ratios (ORs) with 95% confidence intervals (CI) excluding one, were considered statistically significant. All analysis were performed in R statistical software version 4.0.2 (R Core Team, Vienna, Austria) [42]. Mediation analyses were performed using an R package medflex [43]. DAGitty, an online-based platform, was used to formally evaluate causal associations [44].

## 3. Results 

Table 1 demonstrates the maternal and neonatal characteristics of our sample population. There were 160 (59.9%) of placentas with PA alone vs 107 (40.1%) with PA and additional placental pathologies. Examining the full cohort with delivery mode data available, 37% of all placental abruption cases were performed via C-section (97/261) vs 63% (164/261) vaginally. Of data available for placental localization, 62% (68/110) of all placental abruption cases from the full cohort presented with marginal localization vs 38% (42/110) with central localization. Briefly, women in both groups were around 30 years of age and had similar lifestyle behaviours, though a higher percentage of hypertensive diseases of pregnancy were reported in placentas with additional pathologies vs PA alone (20.6% vs 8.6%, respectively). Babies were born in their third trimester (Table 1). Out of the full cohort, 58% (156/267) of babies were born prematurely (gestational age < 37 weeks). 

**Figure 1:**
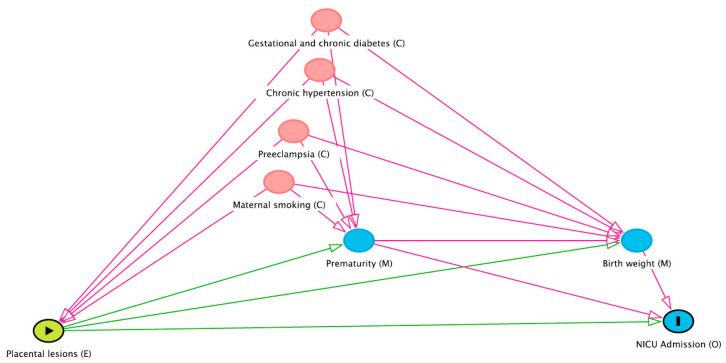
Directed Acyclic Graph (DAG) showing the assumed confounding effects of maternal smoking, preeclampsia, chronic hypertension, and gestational or chronic diabetes with the mediators of prematurity and birthweight between placental lesions and NICU admission. E—exposure; O—outcome; C—confounder; M—mediator.

After listwise deletion (*n* = 37, 13.9%), 142 (61.7%) placentas with PA alone and 88 (38.3%) placentas with additional pathologies remained for the analysis of primary outcome, NICU admission. NICU admission was more frequent among placentas with additional pathologies (*n* = 68, 77.3%) than placentas with PA alone (*n* = 83, 58.5%). Table 2 depicts the effect of additional pathologies (i.e., placental lesions) on primary outcome, NICU admission. The total effect of placental lesions represents the sum of the direct effect of placental lesions to NICU admission and indirect effects (e.g., acting through prematurity). Figure 1 represents the conceptual model of mediation analysis with direct and indirect pathways from the placental lesions to the primary outcome, NICU admission. Results from the adjusted and unadjusted models indicate higher odds of NICU admission in pregnancies complicated by placental lesions. In the adjusted model OR was 2.37 (1.28–4.52; *p* = 0.01). Indirect effect acting through prematurity was OR 1.79 (95% CI 1.12–2.75; *p* = 0.01) and through birthweight OR 2.12 (95% CI 1.40–3.18; *p* < 0.001). There was no evidence for a significant direct effect in either prematurity (*p* = 0.29) or birthweight (*p* = 0.69) mediated analysis. No evidence was found for exposure-mediator interaction between placental lesions and prematurity (*p* = 0.36) or between placental lesions and birthweight (*p* = 0.56).

Table 3 shows the effect of placental lesions on the diagnosis of small-for-gestational age (<10th percentile), base deficit, and cord pH. Of the records available for analysis, 38.1% of women with pregnancies complicated by placental abruption and lesions gave birth to SGA babies. However, this proportion was very similar to the percentage (37.8%) of women giving birth to babies of appropriate gestational age. More women with pregnancies complicated by placental abruption and placental lesions (80%) gave birth to babies requiring chest compressions and resuscitation than babies who did not require chest compressions and resuscitation (38%). Similarly, more women with such pregnancies gave birth to babies with low Apgar scores (72.7%) vs Apgar > 5 at 10-min (36.9%) (Table 3). 

There was no statistically significant difference in the odds of SGA or having an adverse neonatal BD value among women with pregnancies complicated by placental abruption and additional lesions, versus pregnancies complicated with abruption only (OR 1.01, 95% CI 0.50–2.00, *p* = 0.97; OR 0.88, 95% CI 0.42–1.77, *p* = 0.72). Similarly, there was no difference in having an adverse cord pH value (OR 1.02, 95% CI 0.49–2.04, *p* = 0.97; Table 3).

Interestingly, women with pregnancies complicated by placental abruption and lesions were more likely to have a baby with Apgar score ≤ 5 at 10-min than pregnancies complicated with abruption only, alone (OR 4.56, 95% CI 1.28–21.26, *p* = 0.03). Lastly, there was no difference in the need for newborn resuscitation between the two groups (OR 6.6, 95% CI 0.95–129.74, *p* = 0.09; Table 3). 

## 4. Discussion 

This study sought to compare the odds of adverse, neonatal outcomes in women with isolated placental abruption vs placental abruption with underlying placental pathologies. There is an added concern when placentas of pregnant women with placental abruption show MVM, FVM, and VUE, as evidence suggests compromised fetal outcome [17,18,19,20]. Based on our study findings, we conclude that women with pregnancies complicated by placental abruption and co-occurring lesions are more likely to have a baby admitted to the NICU and a low Apgar score at 10-min than pregnancies with placental abruption alone. Moreover, these differences were mostly mediated through prematurity and birthweight.

In the adjusted and unadjusted analysis, we found a significant association between pregnancies complicated by placental abruption and pathologies to NICU admission. Our mediation analyses suggest that these effects act through prematurity and birthweight; however, future studies are needed to validate our finding. Neonatologists follow strict criteria for NICU admission, including evaluating the full clinical picture of the neonate. Specifically, gestational age and weight are factors they consider during admission. Studies demonstrate that maternal health history (i.e., smoking and prior placental abruption diagnosis) can impact subsequent risk of placental abruption [4,45,46]. Therefore, if the neonatologist knows the clinical history of the mother in addition to gestational age and weight, these factors together could potentially influence their decision to admit. 

The presence of underlying placental lesions, as well as placental abruption, are suggested to be associated with a diverse range of neonatal problems, including lower Apgar scores and neonatal asphyxia. Specifically, lower Apgar scores at 1 and 5 min are associated with the placental lesions of maternal vascular malperfusion and intrauterine infection [47,48]. Beebe et al. determined a relationship between a high rate of chorioamnionitis in term and premature infants and low Apgar scores at 1-min [47]. Lower Apgar scores might loosely indicate other respiratory outcomes, such as neonatal RDS or neonatal asphyxia [49]. Prevalent lesions associated with neonatal asphyxia are typically vascular in nature, referring to chorioamnionitis with fetal vasculitis and fetal thrombotic vasculopathy [50,51]. 

Interestingly, reports in the literature show that placental lesions are associated with decreased incidence of respiratory distress syndrome (RDS) [45,49]. Specifically, the incidence of RDS is lower in infants exposed to chorioamnionitis (ORs 0.1–0.6 95% CI 0.02–0.8) [52,53,54]. The biological mechanism behind placental inflammation in RDS stems from increased amounts of interleukin-1 beta (IL-1*β*) in chorioamnionitis. IL-1*β* stimulates corticotrophin-releasing factor and corticotrophin [55,56,57]. Production of these hormones elevates cortisol production, which accelerates lung maturation and decreases RDS incidence [57]. Moreover, the lung mesenchymal tissue decreases, while the epithelial surface area and airspace lung volume increase. This leads to mature lung development and supports gas exchange [54,58,59]. In contrast, very preterm infants exposed to chorioamnionitis were at higher risk for bronchopulmonary dysplasia (BPD), due to elevated levels of IL-8, granulocyte colony-stimulating factor, and anti-inflammatory IL-10 after adjusting for duration of gestation and severity of respiratory distress during the first day after birth [60]. 

There is less focus in the literature on exclusively Apgar scores. In fact, only few publications have linked placental abruption to relatable outcomes, such as increased acidosis risk (a marker of hypoxia) and elevated need for resuscitation [61,62,63]. The increased risk in these outcomes is affected by the interaction between placental abruption status, preterm birth, hypoxia, and blood loss (i.e., fetomaternal hemorrhage) [64]. 

A low Apgar score at later intervals could be used as a proxy for neurological impairment, including cerebral palsy [65]. Recent studies suggest a strong correlation between low Apgar score and cerebral palsy in children born to term or with normal birth weight [66,67]. In particular, among children with a birth weight of 2500 g or more, those with an Apgar score of less than 4 at 5-min were more likely to have cerebral palsy than children who had an Apgar score of more than 8 (OR 125, 95% CI 91 to 170) [65]. In placental abruption cases, evidence supports the impact of immaturity, birthweight, gestational age, and malformations of the central nervous system decreasing Apgar scores, potentially contributing to respiratory difficulties, and possibly increasing the risk for cerebral palsy [68,69,70]. Furthermore, there is increasing evidence showing maternal infection causing fetal inflammation, leading to neonatal brain injury [48]. In particular, inflammatory cytokines are neurotoxic in vitro [71] and in vivo [72] and are shown to inhibit oligodendryocytes in the developing white matter [73]. Histological chorioamnionitis and neonatal blood inflammatory cytokines are likely significantly higher in infants with cerebral palsy [74,75]. Hence, the inflammatory environment at birth plays a role in neonatal brain injury. 

### 4.1. Strengths & Limitations

There are numerous strengths of this study. Unlike previous placental pathology literature, we adjusted for known confounders (maternal smoking, maternal hypertension/preeclampsia, maternal diabetes) during our NICU admission analysis. Moreover, we followed thorough statistical approaches to test if birthweight and prematurity were mediators. We separated our neonatal outcomes, rather than combining them in order to tailor specific recommendations to pathologists and neonatologists. Fortunately, our robust sample size permitted us to examine outcomes in this manner. Data entry was performed by two extractors and consequently validated. However, there remains a possibility of misclassification bias when organizing the placental pathologies, as well as missing data or incompleteness on EMRs for SGA. Further analysis of outcomes of interest such as IVH could not be performed due to insufficient numbers. 

### 4.2. Future Directions 

A future study is underway to examine identical outcomes in connection with the specific pathologies of interest. Chorioamnionitis with respiratory outcomes and Apgar scores will be explored in detail. Another notable endeavor is to develop a national registry of prospectively collected data, to pool rare neonatal outcomes such as IVH across institutions and thus increase sample size. Assessment of coexisting placental pathology(ies) in pregnancies complicated with abruption and its potential impact on infant developmental outcome (i.e., cerebral palsy) might be warranted.

## 5. Conclusions 

Coexisting placental pathology(ies) could potentially impact acute neonatal outcomes, such as NICU admission and Apgar scores in pregnancies complicated by PA. However, future multi-institutional studies of robust sample sizes are needed to ascertain these findings to greater certainty. This pilot study can be replicated by other institutions to determine if obstetrical and neonatal teams should mobilize resources in anticipation of the need for neonatal resuscitation. Since placental pathologies such as VUE may recur in certain cases of subsequent pregnancies [38], our next step is to examine neonatal risks by placental pathology type to inform future neonatal delivery interventions. 

## Figures and Tables

**Table 1:** Characteristics of the Cohorts (*n* = 287).

Maternal Demographics	Placentas with Placental Abruption Only (*n* = 160, 59.9%)	Placentas with Additional Pathologies (*n* = 107, 40.1%)	*p* Value
Maternal age (mean, SD)	31.1 (6.1)	30.4 (5.3)	0.20
Parity (mean, SD)	0.5 (3.0)	0.3 (0.6)	0.68
Pre-pregnancy BMI value (mean, SD)	25.1 (6.0)	25.6 (6.5)	0.94
Diabetes (*n*, %)	24 (15.0)	7 (6.5)	0.05
Previous smoker (*n*, %)	25 (16.6)	11 (11.0)	0.27
Previous history of abruption (*n*, %)	10 (7.7)	4 (4.7)	0.42
Chronic hypertension, Gestational Hypertension or preeclampsia (*n*, %)	13 (8.6)	20 (20.6)	0.01
Other medical conditions (Pregestational diabetes, gestational diabetes, thrombophilia) (*n*, %)	111 (71.2))	67 (66.3)	0.49
**Neonatal Demographics**			
Gestational age in weeks (mean, SD)	35.7 (4.2)	33.1 (5.6)	<0.001
Birthweight (g) (mean, SD)	2638.1 (793.9)	2015.7 (967.9)	<0.001
Sex (Female, %)	68 (43.6)	53 (51.5)	0.25

BMI, body mass index; SD, standard deviation.

**Table 2:** Placental lesions and neonatal intensive care unit (NICU) admission.

Term	OR (95% CI)	*p* Value
Unadjusted logistic regression		
Placental lesions	2.42 (1.34, 4.48)	0.004
Adjusted logistic regression		
Placental lesions	2.37 (1.28, 4.52)	0.01
Maternal Smoking	3.45 (1.33, 10.77)	0.02
Maternal Hypertension/Preeclampsia	1.58 (0.65, 4.29)	0.33
Maternal Diabetes	0.31 (0.13, 0.73)	0.01
Mediation analysis; mediator = Prematurity ^a^		
Natural Direct Effect	1.32 (0.78, 2.19)	0.29
Natural Indirect Effect	1.79 (1.12, 2.75)	0.01
Total Effect	2.37 (1.17, 4.51)	0.01
Mediation analysis; mediator = Birthweight		
Natural Direct Effect	1.12 (0.62, 1.99)	0.69
Natural Indirect Effect	2.12 (1.40, 3.18)	<0.001
Total Effect	2.38 (1.19, 4.63)	0.01

^a^ Gestational age < 37 weeks.

**Table 3:** The effect of placental abruption and underlying lesions on perinatal outcomes.

Perinatal Outcome	Placental Lesions (*n*, %)	OR (95% CI)	*p* Value
SGA ^a^ (*n* = 42)	16 (38.1)	1.01 (0.50–2.00)	0.97
Appropriate weight ^b^ (*n* = 188)	71 (37.8)		
BD 10–15.9 or BD 16 (*n* = 42)	14 (33.3)	0.88 (0.42–1.77)	0.72
Normal BD (*n* = 171) ^c^	62 (36.3)		
Cord pH 7 or 7.1–7.15 (*n* = 42)	15 (35.7)	1.02 (0.49–2.04)	0.97
Normal cord pH (*n* = 164) ^d^	58 (35.4)		
Apgar 5 at 10-min (*n* = 11)	8 (72.7)	4.56 (1.28–21.26)	0.03
Apgar > 5 at 10-min (*n* = 225)	83 (36.9)		
Need for resuscitation (*n* = 5) ^e^	4 (80.0)	6.57 (0.95–129.74)	0.09
No need for resuscitation (*n* = 230)	87 (37.8)		

^a^ Small-for-gestational age < 10th percentile; ^b^ 10–90th percentile; ^c^ Base deficit < 10; ^d^ Cord pH > 7.16; ^e^ Need for chest compressions and/or epinephrine in the delivery room. SGA, small-for-gestational age; BD, base deficit; OR, odds ratio; CI, confidence interval.

## Data Availability

The data presented in this study are available on request from the corresponding authors.

## Supplementary Materials

The following are available online at https://www.mdpi.com/article/10.3390/jcm10235693/s1, Placental lesion definitions.
