# Supplementary material for: Impact of Co-Existing Placental Pathologies in Pregnancies Complicated by Placental Abruption and Acute Neonatal Outcomes
Source: J Clin Med. 2021 Dec 3;10(23):5693. doi: 10.3390/jcm10235693 (PMC8658381; doi:10.3390/jcm10235693)
Supplement: Supplementary file 1 [file jcm-10-05693-s001.zip › jcm-1482939-supplementary.pdf]

Supplementary Material (S1) Placental lesion definitions

Lesion Definitions

MVM was defined as the presence of at least two of the following features, provided that one feature was accelerated villous maturation away from infarction (#3), or decidual vasculopathy (#4)

MVM definition

1) placental weight < 10th percentile;

2) infarction;

3) accelerated villous maturation away from infarction; and

4) decidual vasculopathy

FVM definition

FVM was defined as thrombosis or avascular villi on their own, or any two features of thrombosis, avascular villi, or intramural fibrin deposition.

VUE definition

Any placenta presenting with features of MVM or FVM in addition to chronic villitis was categorized as villitis.
